# Association between the Regulator of G-protein Signaling 9 Gene and Patients with Methamphetamine Use Disorder and Schizophrenia

**DOI:** 10.2174/157015911795017029

**Published:** 2011-03

**Authors:** Y Okahisa, M Kodama, M Takaki, T Inada, N Uchimura, M Yamada, N Iwata, M Iyo, I Sora, N Ozaki, H Ujike

**Affiliations:** 1 Department of Neuropsychiatry, Okayama University Graduate School of Medicine, Dentistry and Pharmaceutical Sciences, Okayama, Japan; 2 JGIDA (Japanese Genetics Initiative for Drug Abuse), Japan; 3 Seiwa Hospital, Tokyo, Japan; 4 Department of Neuropsychiatry, Kurume University Graduate School of Medicine, Kurume, Japan; 5 Department of Psychogeriatrics, National Institute of Mental Health, National Center of Neurology and Psychiatry, Kodaira, Japan; 6 Department of Psychiatry, Fujita Health University School of Medicine, Houmei, Japan; 7 Department of Psychiatry, Chiba University Graduate School of Medicine, Chiba, Japan; 8 Department of Neuroscience, Division of Psychobiology, Tohoku University Graduate School of Medicine, Sendai, Japan; 9 Department of Psychiatry, Nagoya University Graduate School of Medicine, Nagoya, Japan

**Keywords:** Substance abuse, methamphetamine, regulator of G-protein signaling 9, case-control association.

## Abstract

The regulator of G-protein signaling (RGS) modulates the functioning of heterotrimeric G protein. RGS9-2 is highly expressed in the striatum and plays a role in modulating dopaminergic receptor-mediated signaling cascades. Previous studies suggested that the RGS9 gene might contribute to the susceptibility to psychotic diseases. Therefore, we investigated the association between the RGS9 gene and two related dopamine psychoses, schizophrenia and methamphetamine use disorders. The subjects comprised 487 patients of schizophrenia and 464 age- and sex-matched healthy controls and 220 patients of methamphetamine use disorder and 289 controls. We genotyped two nonsynonymous polymorphisms, rs12452285 (Leu225Ser) and rs34797451 (His498Arg), of the RGS9 gene. Rs34797451 showed monomorphism in the present Japanese population, but rs12452285 showed polymorphism. There were no significant differences in genotypic or allelic distributions of rs12452285 between patients with schizophrenia and the corresponding control or between patients with methamphetamine use disorder and the corresponding control. We also analyzed the clinical features of methamphetamine use disorder. We found a significant association in allelic distribution with the phenotypes of age at first consumption (p=0.047). The present study suggested that the RGS9 gene is unlikely to play a major role in schizophrenia and methamphetamine dependence liability and/or the development of methamphetamine induced psychosis, at least in a Japanese population.

## INTRODUCTION

Human and animal studies suggest that the D2-like dopamine receptors play a central role in the development of substance dependence and substance-induced psychotic disorders due to consumption of a diverse class of drugs, e.g., alcohol, nicotine, opioids, cannabinoids, cocaine, and amphetamines, and also of endogenous psychosis of schizophrenia [1-3]. Two animal models of schizophrenia, an amphetamine-sensitized rat and the phencyclidine-treated rat, showed marked dopamine supersensitivity and an increase in the proportion of striatal D2 receptors in the high-affinity state [4-7]. These findings indicate that the dopamine system is involved in the neural mechanisms of psychiatric disorders, substance use disorders, and schizophrenia.

The activity of the dopamine receptors is regulated by intricate mechanisms including the heterotrimeric G protein system. The regulator of G-protein signaling (RGS) modulates the functioning of heterotrimeric G protein in part by stimulating the GTPase activity of the G protein  subunits [8, 9]. RGS9 is a member of the RGS family, and the RGS9 gene gives rise to two products, RGS9-1 and RGS9-2, *via* alternative splicing [10, 11]. RGS9-1 is expressed in the retina, and RGS9-2 is highly expressed in the striatum and plays a role in modulating dopaminergic-mediated signaling cascades [12, 13]. Previous studies showed that the expression of RGS9-2 in the postmortem brain of schizophrenia patients was lower than that of controls [14, 15]. RGS9 knockout mice showed supersensitivity to dopamine and marked elevation in the proportion of D2 high-affinity receptors [13]. These findings suggest that the RGS9 gene variation contributes to the sensitivity of D2 receptors in the brain and to development of psychotic disorders.

It is well known that consumption of methamphetamine, an indirect dopamine agonist, produces psychosis at a high rate. The symptoms of methamphetamine-induced psychosis are similar to those of schizophrenia, and the diseases show a cognate course of illness. Thus, it seemed that methamphetamine use disorder and schizophrenia share in part the mechanisms of their neural pathogenesis. Based on the above rationale, we investigated the association between the RGS9 gene and methamphetamine use disorder or schizophrenia in a Japanese population.

## METHODS

### Subjects

The subjects comprised 220 unrelated patients with methamphetamine dependence (175 males and 45 females, average age 37.0 ± 11.8 years) who met the ICD-10-DCR criteria (F15.2), 486 schizophrenic patients (247 males and 240 females, average age 50.5 ± 12.8 years) who met F20, and two sets of corresponding age-, sex-, and geographical origin-matched healthy controls, 289 control subjects (225 males and 64 females, average age 37.1 ± 12.8 years) and 464 control subjects (225 males and 239 females, average age 51.3 ± 14.3 years) . Two hundred and eighteen of the patients with methamphetamine dependence have or had comorbid methamphetamine psychosis (F15.5). Among the schizophrenic patients, 221 were the paranoid subtype and 239 were the hebephrenic subtype. Diagnosis of methamphetamine use disorder and schizophrenia and determination of subtype was performed by two trained psychiatrists on the basis of all available information. Most of the control subjects were medical staff members who had no past history or family history of substance dependence or major psychotic disorders. All subjects were Japanese, born and living in restricted areas of Japan. This study was initiated after receiving the approval of the ethical committees of the participating institutions. Written informed consent was obtained from all participants.

### Clinical Phenotypes

Clinical observation has revealed substantial inter-individual differences in certain phenotypes of methamphetamine-taking behavior and psychosis that seem to be regulated, at least in part, genetically. The rationale and methods of the subgrouping of phenotypes were previously described [16]. In brief, the patients with methamphetamine dependence and psychosis were divided into five subgroups according to the following clinical phenotypes: multisubstance-abuse status, age at first consumption of methamphetamine, latency to the onset of psychotic symptoms after the first consumption of methamphetamine, prognosis of psychosis after therapy, and the complication of spontaneous relapse to a psychotic state.

### Genotyping

Peripheral blood was obtained from the subjects, and the genomic DNA was extracted from peripheral leukocytes using a standard procedure. We selected two nonsynonymous polymorphisms, rs12452285 (Leu225Ser) in exon 12 and rs34797451 (His498Arg) in exon 18, of the RGS9 gene. Genotyping was performed using TaqMan technology on an ABI7500 Real Time PCR system (Applied Biosystems, Foster City, CA, USA). All genotyping was performed in a blinded fashion, with the control and case samples mixed randomly.

### Statistical Analysis

Statistical analysis of association was performed using SNPAlyze software (Dynacom Co., Mobara, Chiba, Japan). Deviation from Hardy-Weinberg equilibrium and the case-control study were tested using the χ^2^ test. The differences between subgroups were evaluated on Fisher’s exact test. Statistical significance was set at 0.05.

## RESULTS

Rs34797451 showed monomorphism in the present Japanese population, but rs12452285 showed polymorphism. Accordingly, subsequent analyses were done on rs12452285 only. The genotype distribution and allele frequencies for the polymorphism of patients with methamphetamine use disorder and control subjects are shown in Table **1** and those for the patients of schizophrenia in Table **2**. The genotype distributions of patients and control subjects did not deviate from Hardy-Weinberg equilibrium at the polymorphism. We found no significant differences in genotypic or allelic distribution of rs12452285 between methamphetamine use disorder and the corresponding controls or between schizophrenia and the corresponding controls.

To investigate further the roles of the RGS9 gene in the pathophysiology of psychosis and drug-taking behaviors, we examined the association of the RGS9 gene with several clinical phenotypes of methamphetamine dependence and psychosis, i.e., the age at first consumption of methamphetamine, latency to onset of psychosis after abuse, prognosis of psychosis after therapy, spontaneous relapse even without reconsumption of methamphetamine, and multiple substance abuse status, which show individual variation and may in part be regulated genetically. There was a significant difference in allelic distribution between the two subgroups divided by age of first use of methamphetamine (p=0.047, Table **3**) but not in genotypic distribution. For the other clinical phenotypes, there was no significant association with genotypic or allelic distributions of rs12452285 of the RS9 gene.

## DISCUSSION

The present study showed that the RGS9 gene is not associated with susceptibility to methamphetamine use disorder or schizophrenia. There was a significant difference in allelic distribution in the phenotype of age at first consumption of methamphetamine (P=0.047), but the significance was marginal, and there was no significant difference in its genotypic distribution, indicating a necessity for confirmation by replication analyses.

It was well known that three different conditions, psychostimulant-induced behavioral sensitization in rodents, psychostimulant-induced psychoses in humans, and chronic schizophrenia show similar longitudinal alternations, progressively enhanced susceptibility to abnormal behaviors, psychotic state, and relapse [17]. Many studies suggest that the susceptibility to dopamine release that developed in the striatum and accumbens is the most direct and common mechanism for the behavioral sensitization phenomenon in rodents after administration of psychostimulants including methamphetamine, amphetamine, and cocaine [18-22]. Two independent groups found approximately twice as much amphetamine-induced dopamine efflux in the striatum of patients with schizophrenia in comparison to healthy controls [23, 24]. These data support a hypothesis that a process of endogenous sensitization of dopaminergic systems is involved in the pathogenesis of schizophrenia.

There are two splice isoforms of the RGS9 gene. RGS9-1 is the short splice isoform and expressed in the retina, and RGS9-2 is the long splice isoform, which is highly expressed in striatum [10, 11]. RGS9-2 was present in a large fraction of D2 receptor-containing neurons and co-expression of RGS9-2 accelerated D2-mediated channel activation [13]. It was revealed that a single injection of amphetamine reduced the RGS9 mRNA levels [25]. RGS9 knockout mice showed heightened locomotor and rewarding responses to cocaine and a marked increase in the proportions of D2^High^ receptors [4, 13]. In particular, RGS9 knockout mice showed conditioned place preference after lower doses of cocaine than the wild type [13]. Consistent with the result, overexpression of RGS9-2 induced by injection of a herpes simplex virus reduced the dopamine sensitivity [13]. The other group reported that mice lacking RGS9 show a 10-fold increase in sensitivity to the rewarding effects of morphine [26]. These lines of evidence suggest that RGS9 plays an important role in substance use disorder by modulating the dopamine pathway *via* D2 receptors. Recently, Seeman *et al.* reported that expression of RGS9-2 was reduced by 22% in the hippocampus of humans with schizophrenia [15], and the result is consistent with the previous study that reported a 40% reduction of RGS9 in the prefrontal cortices in schizophrenia [14]. They also found that the expression of RGS9-2 in the amphetamine-sensitized rat striatum was reduced by 11% [15].

The RGS9 gene is located on chromosome region 17q21-25 [11], and the region was implicated in major mental illness susceptibility through linkage studies [27-30]. We analyzed two SNPs, rs12452285 (Leu225Ser) and rs34797451 (His498Arg), of the RGS9 gene because these are the only two nonsynonymous polymorphisms in the RGS9 gene registered in the NCBI SNP database. Recently, Liou *et al.* examined a possible association between the RGS9 gene and tardive dyskinesia using seven single nucleotide polymorphisms, rs8077696, rs8070231, rs2292593, rs2292592, rs9916525, rs1122079 and rs4790953. In haplotype analyses, they found a significant association with the haplotype consisting of rs8077696, rs8070231, and rs2292593 of the RGS9 gene [31]. Tardive dyskinesia is an abnormal involuntary movement disorder usually caused by chronic antipsychotic treatment, which blocks D2 dopamine receptors and may induce subsequent supersensitivity of D2 receptors. Therefore, it is possible that the haplotype consisting of these markers is associated with functional changes in RGS9, and a possible candidate for RGS9 analyses for methamphetamine use disorder or schizophrenia.

In conclusion, this study showed that the RGS9 gene is unlikely play a major role in schizophrenia and methamphetamine use disorder liability and/or the development of methamphetamine induced psychosis, at least in a Japanese population.

## Figures and Tables

**Table 1.** Genotype and Allele Distributution of rs12452285 of the RGS9 Gene with METH Use Disorder and Controls

			Genotype(%)		*P*	Allele(%)		*P*
rs12452285	N	C/C	C/T	T/T		C	T	
Controls	289	266(92.0)	23(8.0)	0(0.0)		555(96.0)	23(4.0)	
Patients	220	200(90.9)	18(8.2)	2(0.9)	0.27	418(95.0)	22(5.0)	0.43

**Table 2.** Genotype and Allele Distribution of rs12452285 of the RGS9 Gene in Patients with Schizophrenia and Controls

			Genotype(%)		*P*	Allele(%)		*P*
rs12452285	N	C/C	C/T	T/T		C	T	
Controls	464	422(91.0)	39(8.4)	3(0.6)		883(95.2)	45(4.8)	
Schizophrenics	487	428(87.9)	57(11.7)	2(0.4)	0.02	913(93.7)	61(6.3)	0.18
Paranoid type	221	190(86.0)	30(13.6)	1(0.4)	0.11	410(92.8)	32(7.2)	0.07
Hebephrenic type	239	215(90.0)	23(9.6)	1(0.4)	0.81	453(94.8)	25(5.2)	0.76

**Table 3.** Subgroups of METH Dependence and Psychosis by Clinical Characteristic

Clinical Phenotype			Genotype(%)		*P*	Allele(%)		*P*
rs12452285	N	C/C	C/T	T/T		C	T	
Multisubstance abuse								
Yes	155	141(91.0)	14(9.0)	0(0.0)	0.06	296(95.5)	14(4.5)	0.34
No	59	53(89.8)	4(6.8)	2(3.4)		110(93.2)	8(6.8)	
Age at first consumption								
≤20	117	103(88.0)	13(11.1)	1(0.9)	0.08	219(93.6)	15(6.4)	0.047
>20	101	96(95.0)	4(4.0)	1(1.0)		196(97.0)	6(3.0)	
Latency to onset of psychosis								
≤3 years	101	93(92.1)	8(7.9)	0(0.0)	0.33	194(96.0)	8(4.0)	0.36
>3 years	92	83(90.2)	7(7.6)	2(2.2)		173(94.0)	11(6.0)	
Prognosis of psychosis								
Transient	112	104(92.9)	7(6.2)	1(0.9)	0.61	215(96.0)	9(4.0)	0.34
Prolonged	90	80(88.9)	9(10.0)	1(1.1)		169(93.9)	11(6.1)	
Spontaneous relapse of psychosis								
Yes	93	85(91.4)	6(6.5)	2(2.1)	0.21	176(94.6)	10(5.4)	0.75
No	117	106(90.6)	11(9.4)	0(0.0)		223(95.3)	11(4.7)	
